# Prepare: Improving End-of-Life Care Practice in Stroke Care: Insights from a National Survey and Semi-Structured Interviews

**DOI:** 10.3390/healthcare13080848

**Published:** 2025-04-08

**Authors:** C. Elizabeth Lightbody, Clare Gordon, Christopher Burton, Catherine Davidson, Damian Jenkinson, Aasima Saeed Patel, Freja Jo Petrie, Alison Rouncefield-Swales, Nikola Sprigg, Katherine Stewart, Mehrunisha Suleman, Caroline Leigh Watkins, Clare Thetford, PREPARE Study Research Team

**Affiliations:** 1 School of Nursing and Midwifery, University of Central Lancashire, Preston PR1 2HE, UK; cgordon8@uclan.ac.uk (C.G.); clwatkins@uclan.ac.uk (C.L.W.); cthetford@uclan.ac.uk (C.T.); 2 Dean of Health Sciences, University of East Anglia, Norwich NR4 7TJ, UK; chris.burton@uea.ac.uk; 3 University Hospitals Dorset, Bournemouth BH7 7DW, UK; damian.jenkinson@uhd.nhs.uk; 4 Research Facilitation and Delivery Unit, University of Central Lancashire, Preston PR1 2HE, UK; apatel212@uclan.ac.uk; 5 Research and Evaluation, The Institute for Research in Schools, London SW7 5HD, UK; alisonrouncefieldswales@researchinschools.org; 6 School of Medicine, Faculty of Medicine & Health Sciences, University of Nottingham, Nottingham NG8 1BB, UK; nikola.sprigg@nottingham.ac.uk; 7 Lancashire Teaching Hospitals NHS Foundation Trust, Preston PR2 9HT, UK; katherine.stewart@lthtr.nhs.uk; 8 The Ethox Centre, University of Oxford, Oxford OX3 7DQ, UK; mehrunisha.suleman@ethox.ox.ac.uk; 9 Global Studies Center, Gulf University for Science and Technology, Kuwait City 32093, Kuwait

**Keywords:** stroke, end-of-life care, experiences, survey

## Abstract

**Background:** Stroke has high mortality. Challenges in providing end-of-life care include uncertainty among healthcare professionals about when to start care. While generic tools and guidelines exist, which outline components of quality end-of life care, they may not fully address stroke’s unpredictable trajectories, complicating care planning. **Objective:** To enhance understanding of end-of-life care post-stroke. **Methods:** We undertook an explanatory sequential mixed methods approach, including a cross-sectional survey and semi-structured interviews. All 286 United Kingdom (UK) National Health Service (NHS) hospitals providing inpatient stroke care were approached for participation in an on-line cross-sectional survey. The survey of healthcare professionals from UK stroke units was used to map current stroke end-of-life care and models of care. Fourteen staff who completed the survey and agreed to a future interview were purposively selected. The semi-structured interviews with healthcare professionals involved in delivering end-of-life care post-stroke were conducted and interpreted using the Theoretical Domains Framework. We aimed to enhance our understanding of the experiences, expectations, challenges and barriers in providing end-of-life care post-stroke, including effective clinical decision-making. **Results:** Across 108 responding survey sites, 317 responses were received. Results showed a lack of structured tools and approaches, an absence of stroke-specific guidance and variable delivery of end-of-life care post-stroke. Thirteen staff (nurses, occupational therapists, medical stroke consultants, and a speech and language therapist) agreed to be interviewed. The data provided a fuller understanding of the context within which end-of-life care post-stroke is delivered. The varied challenges faced include: uncertain prognosis, complex decision-making process, varying skill levels, staffing levels, the hospital environment, emotional strain on both families and staff, inequitable access to specialist palliative care, and difficulties associated with different models of care (stroke service structures and cultural context). **Conclusions:** Provision of end-of-life care post-stroke is complex, challenging, uncertain, and inconsistent. There is limited evidence or guidance to support healthcare professionals. There is a need for implementation support, which includes education, to better enable quality and more consistent end-of-life care post-stroke. Further research is required to assess interventions that can support end-of-life care post-stroke to aid clinicians in providing quality palliative care for stroke patients.

## 1. Introduction

Stroke is a major cause of death, with 13% of patients dying in hospital (this figure is much higher in some types of stroke [1]) and 25–30% of survivors dying within a year [2]. In England, stroke-related deaths total 32,000 annually [3].

Unpredictable trajectories of stroke complicate care planning [4,5]. The abruptness with severe stroke from normal function to sudden death restricts opportunities for advance care planning. Others may have an erratic trajectory of prologued declines with recovery, meaning the timing of death is less certain than other conditions, such as cancer. All these factors contribute to uncertainty when planning care. The absence of stroke-specific end-of-life care guidelines further complicates management. Unlike cancer, which has well-established palliative care pathways, stroke lacks standardised protocols to guide decision-making in the transition to end-of-life care [6]. As a result, healthcare professionals often rely on generic end-of-life frameworks, which are less able to account for the complexity of stroke and the impact of stroke on symptom management. Many stroke patients also experience communication difficulties, fluctuating consciousness, or cognitive impairments [7,8], challenging shared decision-making. This uncertainty leads to inconsistent care and difficulty aligning treatment with patient and family expectations [9]. An audit in a large NHS Trust highlighted limited access to specialist palliative care, with most discussions occurring with families rather than patients and only two-thirds of patients having individualised care plans.

National Health Service (NHS) and government publications outline what quality end-of-life care should look like [10,11,12,13,14]. The National Clinical Guideline for Stroke [15] makes several recommendations about what should be available, as well as key considerations, but acknowledges gaps in research on implementation. The Guideline [15] is clear that stroke teams must increase their awareness and expertise in end-of-life care and recognise that this is a core part of their role. However, research on end-of-life care in stroke remains limited, as clinical focus is on acute treatment and rehabilitation.

Several studies have described stroke end-of-life care needs [16,17,18,19,20]. An international review on end-of-life care post-stroke [21] reported poor symptom control, insufficient emotional care, family difficulties accessing information about the patient’s condition, and inadequate support. From an organisational perspective, the stroke service structure and cultural context as a place where end-of-life care is delivered, including staffing, skills, and logistical issues, needs to be examined.

Aim: To enhance understanding of the experiences, expectations, challenges, and barriers in providing end-of-life care post-stroke, including clinical decision-making.

## 2. Methods

### 2.1. Design

We undertook an explanatory sequential mixed methods approach, conducting a cross-sectional survey and semi-structured interviews with NHS staff members providing end-of-life care post-stroke. End-of-life care is generally defined as care in the final 12 months of life [22,23,24], but in this study, end-of-life care is defined as care for patients at risk of dying within 30 days of hospital admission post-stroke as 11–30% of people die within 30 days [25]. The study was reviewed by the NRES Committee North West—Greater Manchester South Research Ethics Committee and received a favourable opinion.

### 2.2. Participant Selection

All 286 UK hospitals providing inpatient stroke care were identified through the Royal College of Physicians’ Sentinel Stroke National Audit Programme (SSNAP) and the Scottish Stroke Care Audit. The named hospital contact for the audit was sent an email asking if the hospital was willing to participate. Hospitals were sent three email reminders to confirm participation. Both audits reported 100% participation. When the hospital confirmed willing to participate, researchers contacted the stroke unit coordinator/ward sister/charge nurse or stroke clinical lead for permission to send an email inviting 3–4 stroke clinicians from each site to complete a survey. Eligible staff included physicians, allied health team leader, stroke nurses, and palliative care leads. Ineligible staff were those not directly involved in end-of-life care decision-making or care delivery. An online survey link was provided, with an option for a paper copy. The first section of the online questionnaire contained the study participant information sheet, with checkboxes to confirm they had read and understood the information sheet; they could withdraw at any time and their contact information and responses would be kept confidential. The participant was unable to proceed unless these checkboxes were completed. Consent was implied by completing the survey.

In terms of interview participants, five sites representing different service configurations/taxonomies (size, type, end-of-life care champion/ Clinical lead and access to end-of-life care specialist). Up to three staff who completed the survey and agreed to a future interview were purposively selected based on factors such as seniority and job role to ensure that diverse experiences were represented within this study. A sampling grid was used to ensure representativity, and those willing to participate signed a pre-interview consent form.

## 3. Data Collection

### 3.1. Survey

A bespoke online survey was developed by the research team, reviewed by expert clinicians in the Research Management Group (RMG), and piloted with clinicians to assess question clarity, response options, and participant acceptability. The survey had eight sections and 41 questions covering several areas: respondent, hospital and stroke service characteristics, use of end-of-life guidance, care responsibilities, environment, education, training, and factors influencing end-of-life care in acute stroke. Responses included free-text (qualitative) and categorical (quantitative) data. Completion time was estimated at 30 min. The survey was distributed using Qualtrics.

### 3.2. Interviews

The semi-structured interview guide was informed by the survey findings, with input from the RMG and Patient and Public Involvement Group. The guide was shaped by the Theoretical Domains Framework (TDF) [26] which is a synthesis of theories primarily focussing on behaviour change. The guide was used to explore factors influencing end-of-life care after stroke. The TDF is a synthesis theory which aims to identify influences on health professional behaviour and determinants of behaviour change. related to implementation of evidence-based recommendations The topics covered included staff experiences, decision-making, barriers and facilitators, care models, communication, patient and family involvement, education, staff support, and readiness for change. The interviews were conducted by telephone or online by experienced qualitative interviewers. All interviews were recorded and then transcribed verbatim and de-identified. Demographic data were collected to describe the sample.

### 3.3. Data Analysis

Survey data were analysed using descriptive statistics and reported as counts and percentages using STATA SE version 17.

All interview transcripts were checked for accuracy and imported into NVivo. A coding framework was developed deductively using the TDF. The TDF is an integrative framework of 14 domains which can facilitate comprehensive assessment of the determinants of current and desired behaviours. At least two researchers undertook content analysis [27] on each transcript independently using the Framework method [28]. The stages of the analysis were: (1) familiarisation of the data, (2) coding (coding anything that might be relevant, line-by-line) by at least two researchers, and (3) interpreting the data by at least two researchers. Where text mapped onto more than one TDF domain, it was coded in both; otherwise, it was coded under the domain that best matched the content. The researchers (C.D., C.G., and C.T.) met to discuss the codes against the initial coding framework and refined it until they all felt that their codes were reflected. Minor differences arose in relation to the mapping of codes, particularly when codes mapped to more than one domain. Conflicts were resolved by a fourth researcher with expertise in using the TDF (C.E.L.).

## 4. Results

### 4.1. Survey

Stroke units were approached between January 2021–September 2022. One hundred and twenty-four hospitals agreed to participate (67% of eligible hospitals) and were sent the questionnaires. One hundred and eight hospitals engaged in the survey across 83 NHS Health Boards/hospitals, serving a geographical area (regional variation 50–100%); 317 survey responses were received, with a site response rate of 87% (i.e., a site completed at least one questionnaire) and 72% of sites providing >=3 responses. Key issues with/reasons for non-participation were: backlogs within R&D that prevented governance; staffing shortages in stroke units, and an inability to identify a suitable principal investigator.

The findings of the survey are presented below, in relation to each of the sections of the survey.

1. Demographics and characteristics of person completing the survey

Survey respondents were stroke nurse consultants, stroke specialist nurses, ward sisters or charge nurses, or physicians. Of the respondents, 39% were nurses, 26% physicians, 25% AHPs, 8% stroke unit palliative care champions/leads, and 2% palliative care specialists. The majority were female (71%), with 182 (57%) having over 5 years’ experience in their current role, and 112 (35%) were between the ages of 41 and 50 years.

2. Hospital and stroke service characteristics

The majority 293 (92%) described their hospital setting as acute. The number of stroke-specific beds on the wards varied, with a majority (40%) having between 21–30 beds.

3. The use of end-of-life guidance and tools in acute stroke care

**Figure 1:**
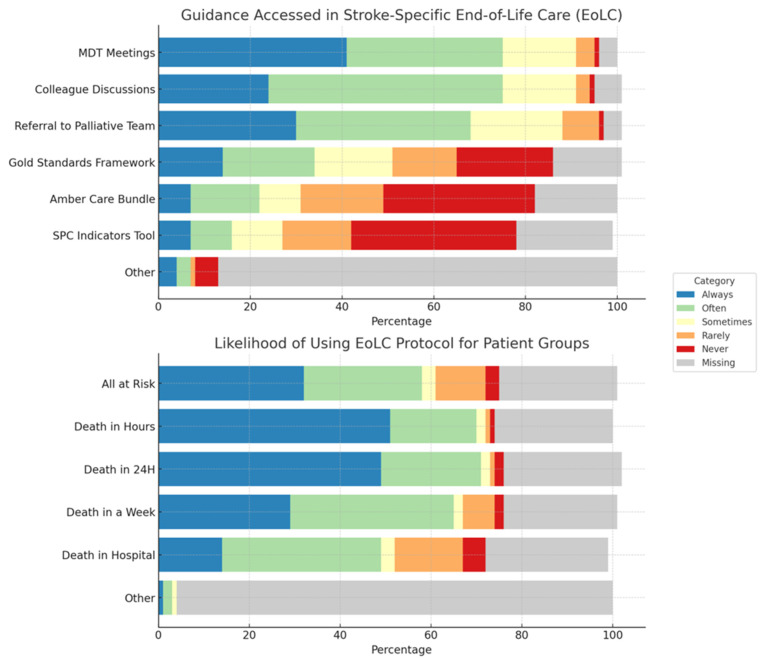
Stacked bar chart depicting use of end-of life care guidance and which patient groups are likely to be managed by an end-of-life care protocol/palliative care team.

The majority of units (69%) used a general end-of-life care protocol for all patients, with only 22 (7%) having a stroke specific end-of-life care protocol and 63 (20%) respondents either being unaware of a protocol or saying they did not have one. Responses indicated that decisions around end-of-life care were frequently supported using multi-disciplinary meetings; unscheduled discussions with ward colleagues; and referral to specialist palliative and end-of-life care teams. A minority of wards used standardised tools to support decision-making, including the Gold Standards Framework, AMBER Care Bundle, and Supportive and Palliative Care Indicators tool (SPICT) (Figure 1)

Overall, use of end-of-life care protocols were initiated mainly when patients were identified as being at risk of dying imminently, with 163 (51%) of respondents reporting they were highly likely initiated protocols when patients expected to die within the coming few hours, and 154 (49%) when patients were expected to die within 24 h. Those identified as being at risk of dying within a week (n = 91, 29%) or expected to die during this hospital admission but after one week (44, 14%) were less likely to have care supported in this way (Figure 1).

Patients most likely to be referred to the palliative care team were those deemed to require specialist palliative care or complex decision making (Table 1).

4. People Responsible for Acute Stroke End-of-Life Care

A total of 41% of respondents had an end-of-life care champion/clinical lead and the majority (n = 277, 87%) had access to a specialist palliative/end-of-life care team. Over half (n = 175, 55%) had out-of-hours specialist palliative/end-of-life care support; however, about a fifth (n = 68, 22%) of respondents were unsure if they had out-of-hours access.

Regarding the members of the MDT most likely to participate in decision-making for end-of-life care in acute stroke patients, involvement varied significantly. Stroke consultants and family members were the most frequently involved, followed by nurses and mid-grade doctors (Table S1). Key decision makers around end-of-life care tended to be all grades of doctors (stroke consultant (89%), junior doctor—foundation and core (64%) mid-grade doctor/specialist registrar (52%)), and the family/carer (61%), with the patient only being involved 38% of the time (see Table S1). The stroke consultant (n = 260, 82%) or mid-grade doctor (n = 171, 54%) were highly likely to communicate decisions around prognosis and end-of-life care to the patient and their significant others. Nurses were the other team members likely to be involved.

5. Where Acute Stroke Patients at the End-of-Life Are Cared for

Patients were most likely to receive end-of-life care in the stroke units (hyper-acute, acute, and rehab), whilst some patients received end-of-life care in their own home or a care home. Generally, respondents felt their ward provided a suitable environment, with adequate peace and privacy for the dying patient (usually n = 186, 59%, sometimes n = 90, 28%), with similar figures reported for family members (usually n = 149, 47%, sometimes n = 110, 34%). About 63% felt they were usually or sometimes able to provide a suitable environment for the family members to stay overnight. Most respondents (n = 222, 70%) could arrange discharge in time for patients who were expected to die within the coming days/weeks and preferred to die at home.

End-of-life care discussions were mainly face-to-face, with some by phone and a few online. Face-to-face conversations usually took place in the relatives’ room or ward office, with some at the patient’s bedside.

6. End-of-Life Care Education and Components of End-of-Life Care

Only 27 (9%) of respondents felt that all staff had the knowledge and skills to provide high quality end-of-life care, and 147 (46%) felt most staff had the knowledge and skills.

The stroke team generally handles direct personal care, MDT communication, and hydration/nutrition management, while symptom assessment, anticipatory prescribing, and psychosocial and spiritual support are shared with the specialist palliative care team. Most respondents (n = 178, 56%) felt there was a procedure for “comfort” or “risk” feeding acute stroke patients receiving end-of-life care, though 20% (n = 62) were unsure (see Table 2). Only a third (n = 97, 31%) reported that stroke patients with end-of-life care needs always or often had an advance care plan. Stroke teams were more likely to discuss end-of-life care and advance care planning with family than the patient. Approximately a third of stroke patients who are conscious, have mental capacity and can communicate (with or without support) are given the opportunity to contribute to an advance care plan, but when the patient is unconscious or lacks mental capacity, 184 (58%) said that they would try to assess the patient’s best interests or preferences in the absence of an advanced decision.

7. Factors Influencing the Provision of End-of-Life Care in Acute Stroke

**Figure 2:**
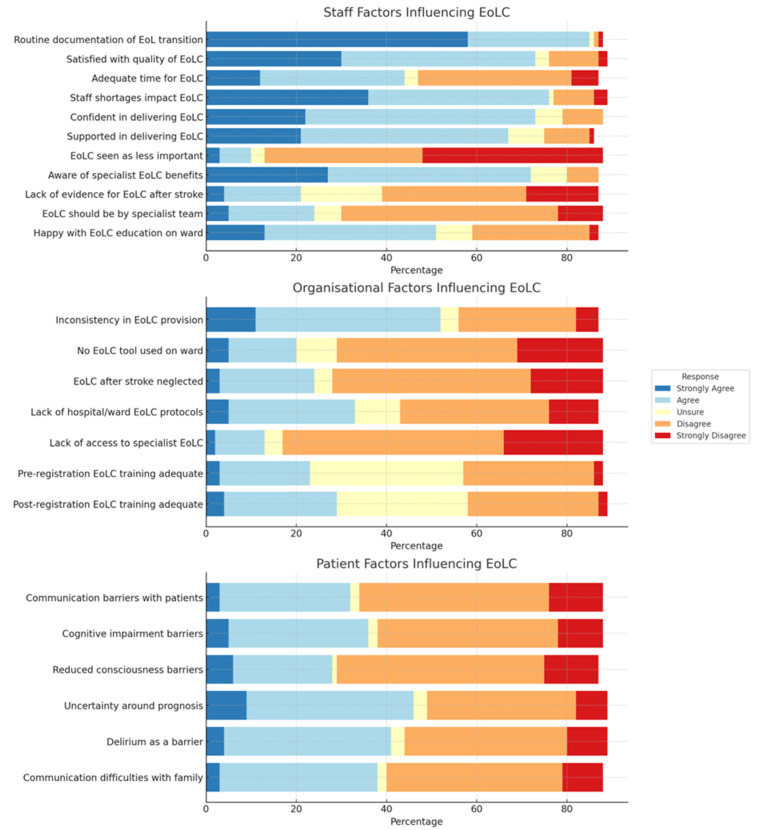
Stacked bar chart depicting staff, organizational, and patient factors influencing provision of end-of-life care in acute stroke.

Staff, organisational, and patient factors influencing end-of-life care provision are presented in Figure 2. Respondents were divided on whether staff had enough time to provide end-of-life care, with most agreeing that staff shortages affected care quality. The majority felt end-of-life care should remain the stroke team’s responsibility, not solely specialist palliative teams.

Organisationally, staff reported good access to specialist palliative/end-of-life care, tools and guidance, but despite this there was reported variability and inconsistency in end-of-life care provision. There was uncertainty about whether pre- and post-registration training for nurses in end-of-life care was sufficient.

Patient factors, such as communication difficulties, cognitive impairment, and consciousness levels, were seen as barriers to quality care. This variability was more pronounced with uncertainty around prognosis, delirium, and communication challenges/disagreements with family and carers.

### 4.2. Interview

Fourteen staff were approached to take part in an interview across five NHS hospitals. A total of 13 participants were interviewed, and one staff member did not respond. In terms of purposive sampling, we achieved a range in terms of stroke unit typology and job role, but all staff interviewed were relatively senior, reflecting those who had agreed to be interviewed. Table 3 presents a description of the interview participants employing hospital. One researcher (AR) interviewed ten participants and a second interviewed three (CT), two of whom took part in a joint interview. Participant characteristics are described in Table 4.

**Figure 3:**
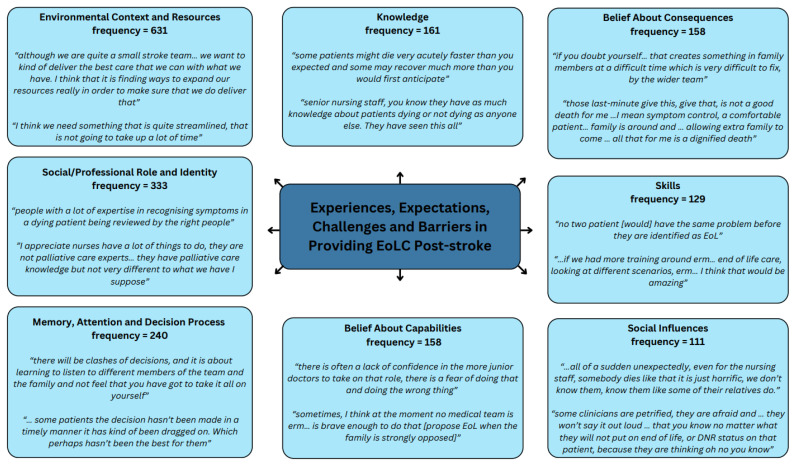
Most frequently coded TDF domains with illustrative quotes.

Within these interviews the TDF domains of “Environmental Context and Resources”, “Social/Professional Role and Identity” and “Memory, Attention and Decision Process” were coded most frequently, accounting for 54% of all references between them. Figure 3 presents the eight most frequently coded domains with illustrative quotes. Themes within the TDF domains and supporting quotes can be found in Table S2.

#### 4.2.1. Environmental Context and Resources

Managing both recovering and end-of-life care patients on the same ward was emotionally challenging for families and staff, as they were antithetical experiences in the same space. Limited space, time, and training hindered patient-centred care. Stroke-specific end-of-life care occurred in stroke or palliative care wards, depending on the Trust, with the ideal setting debated, and dependent on bed availability. Individual rooms offered more dignity but were scarce commodity and usually prioritised for those patients who were imminently going to die.

It was deemed that better and consistent staffing improved support, as interactions with patients/family were more likely to be adequately documented. The involvement of bereavement and palliative care teams were valued and helped free up staff time. Some participants felt experiencing empathy could help prioritise end-of-life care with competing clinical demands.

*“If you don’t have empathy you won’t prioritise”*

(PRE002)

Discharge delays upset families when patients wished to die at home. Staff supported home deaths but faced logistical barriers. End-of-life care discharge processes varied, often delayed by documentation and equipment issues, which meant it was not always achieved. Balancing what feels right with practical needs was difficult.

One hospital had an ‘Emergency Healthcare Plan’ to facilitate patient readmission if home care failed.

*“we have had people that were desperate to get home and we have done everything we could to get everything in place, but unfortunately they passed away before we even got a chance of taking them.”*

(PRE013)

#### 4.2.2. Social/Professional Role and Identity

End-of-life care was delivered by a multidisciplinary team, including specialist palliative care and bereavement staff. Generally, specialist palliative care teams, consultants, and stroke teams worked closely together. However, multidisciplinary engagement varied, with some hospitals lacking a clear process.

*“within this trust there is no proper process of multidisciplinary engagement for end-of-life-care”*

(PRE004)

Speech and language therapists advised on maintaining patient comfort while eating and drinking.

*“advising on what is the least distressing consistency and educating the family and the staff on the ward”*

(PRE006)

Nursing assistants provided hands-on care but lacked palliative training despite strong interest.

*“they (nursing assistants) are showing such an interest in palliative care, and I think they feel quite frustrated that they can’t act on that interest there”*

(PRE008)

Occupational therapists supported functional needs, decision-making, and discharge facilitation before specialist palliative care took over, though some felt their role in stroke end-of-life care needed better understanding.

*“because someone is end-of-life, doesn’t mean that … the person could not be more comfortable … be able to gain more connection with people, or more joy in eating and drinking”*

(PRE011)

In some wards, experienced nurses led end-of-life care due to limited consultant availability. Nurses often wanted more consultant involvement, particularly in family communication, with a liaison role suggested to improve family communication. However, one nurse considered that nurses are experts in palliative care and always at the forefront.

*“we are relatively self-sufficient in that care is quite nurse-led a lot of the time because we don’t have that senior consultant around 5 days a week”.*

(PRE007 and PRE008)

Consultants’ views on their role in end-of-life care varied. Some consultants admitted minimal involvement choosing instead to focus more on acute care, reassured by the skills of their team, especially nurses.

*“I don’t usually talk to families if I think the patient is not going to die within 6 months, I should be but I don’t”*

(PRE009)

#### 4.2.3. Memory, Attention, and Decision Process

Decisions are usually made by a consultant-led MDT. This approach is valued as it helps manage disagreements within the team and encourages listening to team members and families, although some felt a consultant was not always needed.

*“If those conversations are had, and it is clear, then I don’t think it has to be a consultant”*

(PRE007 and PRE008)

Nurses were frustrated when decision-making was unnecessarily protracted or disagreement amongst clinicians or family members prevented the decision from going ahead, resulting in some patients being ‘over-treated’. This was not deemed to be in the patient’s best interest. Staff occasionally made “difficult calls” moving patients against family wishes, believing families should be informed that it is not their decision to make to avoid delays. End-of-life care could overwhelm staff, but teamwork ensured patient and family needs were met.

*“often times the consultants will delay end-of-life-care until all the family are in agreement”.*

(PRE002)

Early end-of-life care discussions were seen as essential, helping meet “spiritual, emotional, and religious” needs. Patients with capacity could be involved in end-of-life care decisions, but often stroke patients are not able to make decisions. One participant shared their timing approach:

*“I don’t do it when they are extremely unwell. When they are stable, when I still do think they are very high risk of having a problem I do discuss it with them”*

(PRE009)

Experienced staff felt more comfortable having end-of-life care discussions, while junior doctors often lacked confidence. Triggers for end-of-life care discussions included airway issues, patient not ‘doing well’, or lack of treatment options. Stroke-related end-of-life care decisions were challenging due to unpredictable outcomes.

*“in stroke the challenge is that sometimes that the suddenness or the acuteness of the stroke makes it a lot more difficult. […] probably patient was not dying last week or when the stroke happened but now that the patient has changed, identifying that probably is one of the, you know something can be improved actually”*

(PRE005)

There was variability in the use of tools to support decision making. Tools reported included the ICARE plan, which was seen as comprehensive covering patient care, family needs, medical review, decision-making, and patient wishes, the NIHSS for measuring severity, and the ICH score to measure blood volume in intracerebral haemorrhage. These scores were considered alongside comorbidities and stroke history. Those who used tools generally felt they had utility.

*“it encourages you each day to identify any issues that you, any needs that you are not meeting … and put a care plan in place for that. And, then it asks you to reflect on the outcome of that as well, how successful that’s been.”*

(PRE007 and PRE008)

However, others noted a lack of tools. One participant felt a tool to predict stroke patient mortality within a certain period would be valuable. The importance of documenting decision-making was also referenced to avoid confusion amongst staff.

#### 4.2.4. Knowledge

Stroke end-of-life care is complex due to unpredictable prognoses. Staff highlighted the need to explain how stroke differs from typical end-of-life care trajectories. More palliative care training was desired but limited by time. Learning was often described as opportunistic.

*“it is the cascade of that information isn’t it, it is like who is at that meeting, and who else learns from it.”*

(PRE006)

Teaching and training are important, but only part of the solution; experience helps with understanding patients’ needs and increases competence.

*“I don’t usually ask my junior doctors to do this discussion, I usually ask them to come to see how I discuss it.”*

(PRE009)

One Trust required all stroke unit staff to complete e-learning resource STARS (Stroke Training and Awareness Resources) competencies, including advanced training for doctors and nurses. Over time, experienced staff found end-of-life care management became ‘second nature.’

*“it comes with experience [….] a lot of what we do nursing wise on the job is from peer learning…”*

(PRE010)

#### 4.2.5. Belief About Capabilities

Staff believed they provided dignified, respectful care and valued the specialist palliative care team. However, care quality varied by staff experience.

*“we do a good job at treating these patients,…providing the dignity and respect that they need and the comfort to the family”*

(PRE003)

Nurses are used to death and felt they had confidence and competence to advocate on behalf of families. However, they felt less confident in having discussions about end-of-life care and wishes.

*‘‘the discussion about where the patient would want to die… that is something we are not good at”*

(PRE009)

Junior doctors lacked confidence due to fear of mistakes (PRE007 and PRE008). It was felt that clear guidance would help raise confidence in making end-of-life care decisions and discussions. Better MDT support and staffing levels could facilitate collaborative end-of-life care decision-making. Stroke’s sudden onset was hard for families to accept, especially when the patient was younger, which led to more family disagreements. Staff reported balancing medical care with family wishes as difficult and best interest meetings were reported as supporting complex decisions, helping to resolve disagreements.

*“no medical team is brave enough to do that” (propose end-of-life-care when the family is strongly opposed)*

(PRE002)

#### 4.2.6. Belief About Consequences

Varying capabilities of individual staff were reported, with junior staff lacking the confidence in communicating with families and making decisions due to fear of getting it wrong. Staff felt that lack of confidence in decision-making could delay end-of-life care, causing distress for families and frustration for nurses, as clinicians are just delaying the inevitable. There were reports that some clinicians may lengthen or change end-of-life care decisions out of fear of making the wrong decision, which was deemed to be emotional for the family, and not in the patient’s best interest.

*“I do feel that I know for sure that some patients the decision hasn’t been made in a timely manner it has kind of been dragged on. Which perhaps hasn’t been the best for them.”*

(PRE006)

*“So those last-minute give this, give that, is not a good death”*

(PRE002)

Doctors were uneasy about communicating end-of-life care decisions, fearing they would upset families further. Wording was key. Fear and lack of confidence of being direct with families affected communication quality, with doubt creating issues that require multiple meetings to resolve.

*“Saying “I’m putting your mum on a pathway” sounds horrible and inhumane”*

(PRE004)

Despite staff hesitations, respondents felt families appreciated honesty. However, families were distressed when end-of-life care decisions were reversed; communication was tricky when end-of-life tools were not used and patients recovered after end-of-life care discussions. It was felt that the Liverpool Care Pathway controversy still affects end-of-life care decisions. Staff reported moving a patient to a ‘bounty bed’ which is a hospital bed that is temporarily made available to accommodate patients, could feel like taking their life. Staff considered that there was a ‘label’ attached to the end-of-life care and felt they had to reassure worried families about hospital tools being used.

*“End-of-life-care or DNR, thinking, oh no, I can’t do that”*

(PRE005)

*“it is more of a tool to make sure that when you come into hospital, we have been doing your observations routinely to make sure that if something goes wrong we can act on it.”*

(PRE004)

There was a feeling that better understanding of end-of-life care would support high-quality care and that training, combined with experiential knowledge will help the MDT understand palliative care.

#### 4.2.7. Skills

Identifying end-of-life care patients relied on highly-skilled staff with experience and MDT input, as there is no universal approach. Some nurses felt skilled enough to manage final stages, especially for elderly patients and where the family were in agreement. Senior staff were recognised for their experience which led to good communication skills. However, it was felt some consultants, registrars, and nurses lacked end-of-life care skills due to low confidence and time constraints.

*“you often have to do it to get it right. And you can listen to someone else doing it…but it is slightly different”*

(PRE004)

Building a rapport with the family and using lay language supported family understanding. Staff emphasised the need for education and experiential learning, as end-of-life care discussions required skill, emotion, and energy

*“a lot of skill… a lot of emotion… a lot of energy’ and that ‘you have got to have the right people who are able to deliver that message”*

(PRE006)

#### 4.2.8. Social Influences

Staff prioritised patient and family wishes for a ‘good death’. However, families could sometimes pressure staff to continue with treatment even though it was not in the patient’s best interest, and staff went along with it to appease them.

*“we have had consultants go, oh well just keep the fluids going because the relative wants them.”*

(PRE010)

Some staff felt that documentation and communication were reinforced through daily safety briefs, ensuring senior nurses and the wider team were aware of and addressed issues, ensuring better MDT collaboration and improved patient care, but this required collective action to implement.

*“we need collective work to improve patient care but often that is difficult. We need to set it all up.”*

(PRE004)

However, one participant noted a hospital culture where death was an unmentionable topic.

*“it is still you know it has got to be the most taboo subjects in hospital still [….] we can’t talk about somebody dying”*

(PRE008)

#### 4.2.9. Emotion

Managing end-of-life care was emotionally challenging, especially when staff had to support both the patient and their family. Staff acknowledged the challenge of managing family’s emotions but felt engaging multiple family members helped.

*“if there is more than one family member…there are different emotions in the room…it just helps manage the situation more effectively.”*

(PRE011)

The busy nature of acute wards and bed shortages often led to staff feeling overwhelmed and conflicted. Patient distress affected staff deeply, with one nurse stating,

*“I hate it when patients are distressed in a bay, sometimes I feel when they know that they are dying I don’t want them to even have any awareness sometimes.”*

(PRE001)

The emotional impact could be long-lasting, especially when care was deemed to have gone “wrong”. Sudden deaths were described as “horrific” and “very emotional” for nursing staff, yet nurses felt compelled to continue working despite the recent loss.

*“we still talk about it now… that case will always stick with me”*

(PRE001)

Most stroke end-of-life care teams lacked official psychological support; instead, support often came from peers or hospital well-being services. One Trust had a psychological support team offering group sessions, initially seen as ‘awkward’ but later as ‘amazing’ and highly valued, though not often used. Another participant mentioned a rare one-off reflection session following a traumatic case, with deaths usually discussed in monthly mortality meetings. Some felt showing emotion demonstrated empathy and improved care.

*“I’ve never had the conversation without crying… it shows you care …I think it makes them feel better”*

(PRE007 and PRE008)

## 5. Discussion

This study has enhanced our understanding of what current end-of-life care post-stroke looks like and the significant challenges that health professionals face in providing compassionate and dignified care. These challenges stem from the uncertain prognosis, complex decision-making process, varying skill levels, staffing levels, the hospital environment, emotional strain on both families and staff, inequitable access to specialist palliative care; and difficulties associated with different models of care.

The multidisciplinary nature of end-of-life care delivery is a crucial finding in this study. Although there was a strong collaboration between stroke and specialist palliative care teams, the lack of clear processes in some hospitals led to inconsistencies in care. These findings align with previous studies that have identified the need for standardised processes and better communication within multidisciplinary teams to ensure consistent and holistic care [29].

Honest, clear, and timely communication around end-of-life care and the potential of death is required to ensure quality care and more informed decisions for patients. Conversations about death and end-of-life care should be started early, but uncertainty about when to initiate end-of-life care after a stroke remains a significant challenge for clinicians. Prognostication in the acute phase is often difficult due to the variable trajectory of stroke recovery, making it hard to determine whether a patient will survive with severe disability or experience further deterioration [30].

Different healthcare professionals faced distinct barriers in making end-of-life decisions for patients following a stroke, with consultants navigating prognostic uncertainty and complex medical decision-making, nurses struggling with prolonged decision processes and emotional burdens, and junior staff lacking confidence due to limited training and experience in end-of-life care. While decisions are generally led by consultants, the role of nurses and other staff in making difficult decisions and communicating with families was also significant. Nurses were often at the forefront of providing care but expressed frustration with prolonged decision-making processes. The emotional and social pressures exerted by family members often complicated decisions, with some staff members feeling compelled to extend treatment to appease families. This often arose when there was a misunderstanding, differences in beliefs or families struggling with the emotional burden of uncertainty [31] or when communication was fractured. For example, it was difficult when families had not accepted their relatives’ imminent death and did not agree with treatment withdrawal or their relative being placed on end-of-life care. This tension between medical recommendations and familial expectations meant that staff sometimes felt they were prioritising relatives wishes over patient wishes. This may stem from debates around the Liverpool Care Pathway where the media portrayed it as “a pathway to euthanasia”, compounded by a deep-rooted reluctance within the UK to address issues around mortality, with hospitals seen as places to heal and prolong life. This aligns with previous research indicating that communication breakdowns, which operate in a complex social context about death and dying, can lead to delays in care and sometimes unnecessary treatment, which may not be in the best interest of the patient [32] and can lead to moral distress among healthcare providers [33]. Better communication about the realities of end-of-life care could help mitigate these tensions and support a more patient-centred approach. One strategy suggested by staff was the use of a liaison role to improve communication with families, which could help bridge the gap between clinical decisions and familial expectations, ensuring that patients receive care that aligns with their best interests.

There was also a lack of consistency and variable quality in the documentation of any conversations had with families’ or patients, meaning that information sometimes did not get effectively communicated. Limited staffing compounded the problem, contributing to delayed decision making, inadequate documentation of patient-family discussions and delayed discharge processes for patients wishing to die at home. Staffing shortages have previously been reported as negatively affecting the quality of communication and the timeliness of end-of-life care decisions [34]. However, the support from specialist palliative care and bereavement teams was viewed as invaluable, assisting in both the logistical aspects of care and providing emotional support for families and staff.

In terms of knowledge and training, this study highlighted training needs across a range of healthcare roles and levels of seniority, including non-professional staff. Staff recognised that stroke end-of-life care requires specific knowledge due to the unpredictable trajectory of the disease, which is often complicated by comorbidities. Consequently, many staff described a need for training around how best to have difficult conversations around end-of-life care with patients and families. Although staff could be taught to have effective discussions with families, this was regarded as a skill learned through experience, so opportunities to learn from more experienced staff should be available. The desire for more formal training, alongside experiential learning, reflects the current understanding in the literature that end-of-life care training must be ongoing, incorporating both theoretical knowledge and practical experience [35].

Additionally, tools such as the ICARE plan, Amber Care, NIHSS, and ICH score, while helpful, were not universally used, indicating a lack of standardised tools to guide decision-making in stroke end-of-life care. Locally developed generic end-of-life care policies and guidance were commonly used. There is evidence to suggest that clinical tools and guidelines can improve the consistency and quality of care, especially in the management of complex, unpredictable cases like stroke [36]. In addition to revealing a lack of use of structured tools and approaches, and an absence of stroke-specific guidance or tools, this work has highlighted huge variation in how end-of-life care is delivered after stroke.

Quality end-of-life care involves multiple components. It is important to ensure that patient needs are met, including symptom control and that patients are treated with dignity. However, this can be challenging in open wards where privacy may not always be provided, and where staff are managing both recovering and end-of-life care patients in the same space. The lack of individualised rooms for end-of-life care patients means that some die in less-than-ideal conditions, with privacy often being sacrificed due to limited bed availability. These observations are consistent with the existing literature that highlights the importance of a dignified death, which can be compromised when patient care spaces are not optimised for end-of-life care [37]. Furthermore, navigating both clinical demands and familial expectations within the same environment often places staff in difficult emotional positions.

The emotional impact on staff, which was especially evident in difficult cases, deeply affected staff, particularly when things were perceived to have gone wrong. However, staff described how there were no formal well-being or psychological support procedures. Support was often provided informally between staff members. A lack of formal debrief or reflection opportunities for staff after patient deaths was also highlighted. The lack of formal psychological support, alongside the emotional demands of the work, reflects a broader issue in healthcare where staff well-being is often overlooked. Peer support, reflective sessions, and institutional programs for staff well-being are critical to maintaining morale and preventing burnout. Moreover, fostering an organisational culture that allows staff to express emotions and seek help when needed is essential for maintaining the quality of care provided [38].

The findings highlight the need for structured policies and systematic training to improve end-of-life care for patients following a stroke. There is a clear need to develop stroke-specific end-of-life care guidelines to address the unique challenges of prognostication, communication, and decision-making, ensuring a more consistent, patient-centred approach that aligns with the complexities of stroke trajectories. Enhanced palliative care training, particularly for nursing assistants and junior doctors, could improve confidence in end-of-life care discussions and prevent unnecessary treatment prolongation. Cultural shifts in hospital settings are needed to normalise conversations around death, ensuring that families receive honest, compassionate communication while prioritising patient dignity and comfort. Addressing these policy and practice gaps could lead to more consistent, timely, and patient-centred stroke end-of-life care. The survey used a self-reporting questionnaire, the assumption being that the responses submitted accurately reflect practice. We tried to mitigate against recall and response bias by having several respondents with different professional backgrounds returning the questionnaire at each hospital. As the survey was not able to provide meaning or context behind responses, we also undertook semi-structured interviews; however, participants self-selected to participate, so there could have been a degree of selection bias. Furthermore, methods to enhance the trustworthiness of the interpretation of the data such as member checking, use of memos, or reflective journaling was not undertaken; however, at least two researchers analysed each transcript, making misinterpretations less likely.

## 6. Conclusions

Despite stroke’s high mortality, there is limited guidance on delivering end-of-life care post-stroke. Variability exists in decision-making, care delivery, and patient/family involvement. End-of-life care after stroke is complex, with challenges like uncertain prognosis, decision-making complexities, inadequate training, emotional distress, and limited staffing. Addressing these challenges requires a multifaceted approach including better training on communication and standardised tools and processes to guide decision-making, while accounting for unique and individual needs of different patients to ensure that patient and family wishes are respected. Prioritising emotional well-being and collaborative multidisciplinary care will improve stroke end-of-life care, ensuring care is compassionate, dignified, and patient-centred. Future research needs to explore the work which needs to be done to implement, embed, and integrate an end-of-life care intervention into everyday practice, providing insight into improving consistency across different healthcare settings.

## Figures and Tables

**Table 1:** Patients referred to specialist palliative and end-of-life care teams.

Which Patients Do You Refer to the Specialist Palliative and End-of-Life Care (EoLC) Team?			
	Yes %	No %	Missing %
None	7	85	8
All patients transitioning to end-of-life care	44	48	8
Patients who require specialist palliative and/or end-of-life care input	65	27	8
Patients who require complex decision making	54	38	8
Patients who require specialist advice on symptoms	64	28	8
Patients who wish to die in their usual place of residence	47	45	8
Patients who wish to die in a hospice	50	42	8
Other	5	87	8

**Table 2:** Who provides different elements of end-of-life care.

Who Provides the Following Elements of Care to Acute Stroke Patients Receiving End-of-Life Care?				
	Stroke Team %	Specialist Palliative and/or End-of-Life Care Team %	Both %	Missing %
Personal care	87	0	3	10
Symptom assessment	36	4	51	10
Symptom management	31	5	54	10
Communicating uncertainty of prognosis	51	3	36	10
Communicating information to the MDT	62	1	27	10
Communicating information to patients	37	1	53	10
Communicating information to those important to the patient	39	1	50	10
Management of hydration and nutrition	63	1	26	10
Anticipatory prescribing	41	4	45	10
Psychosocial support for the patient	34	9	44	12
Psychosocial support for those important to the patient	34	9	45	11
Spiritual support for the patient	32	16	36	16
Spiritual support for those important to the patient	32	18	33	17
Other	3	0	1	96

**Table 3:** Description of the interview participants employing hospital.

Site	Location	Number of Beds in the Unit	EoLC Lead	Specialist EoLC in Hours	Specialist EoLC out of Hours
Acute stroke unit with hyper-acute beds	Urban	21–30 beds	No	Yes	unsure
Comprehensive Stroke Centre (CSC)	City hospital	40+ beds	Yes	Yes	Yes
Acute stroke unit	Rural	1–10 beds	No	Yes	No
Rehabilitation unit	Rural	11–20 beds	Yes	No	No
Integrated acute and rehabilitation unit	Urban	21–30 beds	No	Yes	Yes

**Table 4:** Characteristics of interview participants.

Participant Code	Current Role/s	Length of Current Role (Years)
PRE001	Stroke nurse consultant	10
PRE002	Ward manager	Unknown
PRE003	Occupational therapy team leader in a hyper-acute/acute stroke unit	2
PRE004	Stroke physician	12
PRE005	Stroke nurse consultant	5
PRE006	Speech and language therapist	6
PRE007	Ward sister	16
PRE008	Stroke nurse practitioner	10
PRE009	Stroke physician	2
PRE010	Stroke nurse practitioner	Unknown
PRE011	Occupational therapy team leader	4
PRE012	Occupational therapist in acute stroke and AHP team lead for acute stroke services	2
PRE013	Stroke specialist nurse—integrated unit with HASU beds, acute beds and rehab beds in one site	1

## Data Availability

The original contributions presented in this study are included in the article/Supplementary Material. Further inquiries can be directed to the corresponding author.

## Supplementary Materials

The following supporting information can be downloaded at: https://www.mdpi.com/article/10.3390/healthcare13080848/s1, Table S1: How likely are the following groups to be involved in the decision-making process around end-of-life care for acute stroke patients? Table S2: TDF domains and supporting quotes.
