# Supplementary material for: Prepare: Improving End-of-Life Care Practice in Stroke Care: Insights from a National Survey and Semi-Structured Interviews
Source: Healthcare (Basel). 2025 Apr 8;13(8):848. doi: 10.3390/healthcare13080848 (PMC12027267; doi:10.3390/healthcare13080848)
Supplement: Supplementary file 1 [file healthcare-13-00848-s001.zip › healthcare-3518044-supplementary.pdf]

Domain	Subtheme	Sample quote
Beliefs about capabilities	Confidence	• *'There is often a lack of confidence in the more junior doctors to take on that role, there is a fear of doing that and doing the wrong thing.' (PRE007 & PRE008)*<br>• '…feel confident in manual handling, I feel confident in erm… complicated conversations. And I feel competent in that as well. So I think competence and also confidence I think there is that. I think as far as erm… the wider team I think it is erm… you know I think I can see that I have training needs, and as a team it could be beneficial to sort of iron out everyone's roles within that.' (PRE011)
	Staff experiences and skills	• *'The staff are very good because most of our staff, nursing staff, have been with us for quite a while, erm… and the senior ones especially will be, will flag up patients even before I do, about the patients that they think are not going to do very well' (PRE009)*
	Connection with patient or family	• *'we do a good job at treating these patients and providing the dignity and respect that they need and the kind of comfort to the family' (PRE003)*
	Consistency in processes	• *'the more that we are doing better MDT discussions and involving the bereavement team I think that our confidence is improving and I think we feel that we are doing the, we are more frequently thinking we have done the best for that patient.' (PRE006)*
	Place of dying	• *'Ensuring that the patient first of all is cared for erm… with dignity, and that some times cannot happen on an open ward, and families don't have the room to grieve if they are in a 4 bedded bay. That is very difficult in itself.' (PRE001)*
Beliefs about consequences	Balancing acute care & EoLC	• *'And if we can control the patients' symptoms and get them in the right place whether that be an acute hospital bed, or whether it be erm… in a nursing home or better still a hospice you know if they are going to die, because the people there are trained properly, you know to look after them. Well then if you have to put me on the spot and say what is a good death it is probably that.' (PRE001)*

	Balancing needs and perspectives	• *'…I need to make sure they understand their family prognosis. Then you walk away feeling quite good because the family understands, it gives them that opportunity to come to terms with it, it gives you that opportunity to make onward referrals to the palliative team, even to the psychology team.' (PRE002)*<br>• *'…yes we do want relatives onboard, but sometimes we are definitely treating the relatives and not the patient in death unfortunately.' (PRE009)*
	Co-ordination and consistency of care	• *'continuity of care is really important in terms of predicting trajectories' (PRE004)*
	Fear of getting it wrong	• *'if you didn't know how to communicate with the right information in the right shape and form, if you don't have that confidence like we said if you doubt yourself, when you [24.26] that creates something in family members at difficult time which is very difficult to fix, by the wider team. You have made something which could have gone so smoothly, with the right way you need meeting after meeting after meeting to settle the families after that, because of one thing.' (PRE004)*<br>• '…some clinicians are petrified they are afraid and it is even though they won't say it out loud you can kind of read it all over their face, that you know no matter what they will not put on end of life, or DNR status on that patient, because they are thinking oh no you know.' (PRE005)
	Perspectives of death and dying	• *'…it is always tricky to label because there is a label attached to end of life care pathway. Everyone expects yes this patient is going to die. And is that the case 100% of time, no. No. It might not happen in one month, it might not happen in two months, might not happen in six months. So, erm… that is the tricky one and that is when we don't always end up using end of life pathway.' (PRE009)*<br>• *'The patient, person probably dies and goes, but who has to live with it, they also have to have a good experience.' (PRE005)*

	Nature of stroke	• *'But we never get it right, we never seem to tie it together unless it is a really quick death, erm... then it almost seems, you know doesn't seem to go very wrong because the death has happened quite quickly and the thing about stroke is that it is very acute.' (PRE001)*<br>• *'...although stroke itself might not be the reason they die erm... it plays a big factor in their quality of life and then leading to earlier death than erm... than it was if they hadn't had a stroke.' (PRE009)*
	Clear communication	• *'...communication is key, isn't it you know just that, them being informed, every step of the way. And them knowing that every step of the way that their loved one was a the centre of that care, and everything was done for their loved ones' best interest. That they, were comfortable, and erm... so, yes I think, for me definitely for the family that they would leave the room thinking well we knew what was happening, and erm... nothing else could be done.' (PRE006)*
	Unexpected consequences	• *'...we did all our assessments and we discharged planned for her because she made a miraculous recovery and at that point the family said, I can't take her home because a week ago you told me she was dying.' (PRE012)*
Memory, attention & decision-making processes	Deciding place of death	• *'how do we support these long term who aren't imminently dying but the wishes are not to be moved from the hospital.' (PRE002)*
	EoLC Vs palliative care	• *'in stroke the challenge is that sometimes that the suddenness or the acuteness of the stroke makes it a lot more difficult. [...] when the patient is dying so yes probably patient was not dying last week or when the stroke happened but now that the patient has changed, identifying that probably is one of the, you know something can be improved actually. (PRE005)*<br>• *'if you talk about in stroke care, you do things post decision point, I think the bereavement team support has been erm... massive in terms of they don't discriminate, the strokes patients with someone else, they see everybody on end of life care and the palliative care [43.34: needs], these patients are probably not very different one thing that would be different, among stroke would be to predict prognosis' (PRE004)*

	Family wishes around decision-making	• *'you will find often times the consultants will delay end of life until they have actually erm... spoke to the family, and all the family are in agreement or and if they are having difficulty in accepting them they also have another meeting where the palliative team is there, to offer support' (PRE002)*<br>• *'When we talk about death yes the patient is dying, but that experience is going to be lived by the people who are still alive.' (PRE005)*<br>• *'I think because some things are a medical decision and I think sometimes people or relatives feel like they are being asked to make the decision about certain things. It is actually no, we are not asking you, we are just letting you know where we are up to in this process and actually they aren't going to survive, and yes we want you to be happy with what we are doing but at the same time this is not a medical, this is a medical decision not on you...' (PRE010)*
	Doing what is morally correct	• '...you want to make it a good experience for them as well, don't you, you don't want to traumatise them for the rest of their lives, over the death of their relative but at the same time it has got to be about the patient, it has to be about the care of the patient and not the care of the relative' (PRE010)
	Having the right people to make decisions	• *'then we will have a discussion at the board round, to say erm... you know kind of give our feedback about how drowsy they are or how we have found them in our assessment, the same with the speech and language therapist. And then as a team I suppose the docs will lead and then we will make the decision or they will make the decision if they are end of life,' (PRE003)*<br>• *'These are conversations that should be happening at the right time and I think, the right time is decided by the right people so and that person has to be similar, has to be senior and familiar with what they are dealing with.' (PRE001)*
	Learning from the past	• *'... there will be clashes of decisions, and it is about learning to listen to different members of the team and the family and not feel that you have got to take it all on yourself.' (PRE006)*

		• *'raising the agenda for advance decision making, to consider what holistic health and wellbeing needs would be preferred by a person as well. So that you know even predating stroke or any other condition, that, some things would be considered then you know' (PRE011)*<br>• *'they feel comfortable so that gives you the reassurance as well they have looked at every element of it. So I think within the stroke unit I am very, you know some of my senior nurses my band 6s and band 7s, they are very good at it. Some of the junior ones which comes with experience, you can understand they are very new to the nursing career altogether. But on the whole, I think I would say that I am happy and comfortable with the conversations that they had with me towards the end of life decision.' (PRE005)*
	Openness to consider EoLC	• *'I do discuss it with the patients. But I don't do it when they are extremely unwell. So for example if someone is unable to breathe properly and I am trying to discuss I won't do that. When they are stable, when I still do think they are very high risk of having a problem I do discuss it with them. Well, the way I do it is usually by asking, asking them do they think they are well and erm... I usually tell them there are two possible outcomes, in every person one is they get better and go home, and the other one is erm... they get worse over time. So that is how I bring up these discussions.' (PRE009)*<br>• *'when I talk to the family and I make the decision I clarify to them that I and my team are going to review this decision, every day while the patient is in hospital. Once the patient, and then the palliative care team will review the decision after that. So, as I said stroke the trajectory isn't always one way, there are many things that can happen so we always, I always say to the... that right now how the patient is this is going to happen. However things change, I am here to review those things. So I always keep a door open about the possibility of things changing' (PRE009)*<br>• *'I don't want to give up on the patient too early either so you will monitor or observe the patient for 24 / 72 hours and if no*

		improvement or any further deterioration I would document consider end of life following consultant review' (PRE005)
	Procedures supporting decisions	• *'once an end of life decision has been taken, again like I said it is not just the consultant who makes that decision with the nursing team, the therapist, all of them we have an MDT meeting and once we have decided that the patient is put on end of life we do refer to this end of life team' (PRE005)*<br>• *'you have to weigh up every patient is different you know, you have got to weigh it all up erm... but you know start the talk early, erm... involve other professionals early, and all sing from the same song sheet and always take into account patients' wishes erm... spiritual, emotional, religious etc.' (PRE001)*<br>• *'And I don't think there is always a right or a wrong is there because every patient is different. But having the bereavement team there, as experts, erm... or just not even experts additional support has helped. It has helped that decision making has been less onerous since then.' (PRE006)*
	Resources affecting decisions	• *'it encourages you each day to identify any issues that you, any needs that you are not meeting. And erm... and put a care plan in place for that. And, then it asks you to go back and reflect on the outcome of that as well, how successful that's been.' (PRE007 & PRE008)*<br>• *'You are having that discussion with the families, can we actually move that patient, but they would rather the patient be in hospital. So what do you do then. When there is the pressures of beds as well.' (PRE002)*<br>• *'Very rarely if they are not around then I make a plan in my head I will talk to them but that never happens because you end up doing other things and you forget altogether but then I am confident that the stroke team or the consultant then picks up on that.' (PRE005)*
	Timely decisions	• *'... some patients the decision hasn't been made in a timely manner it has kind of been dragged on. Which perhaps hasn't been the best for them.' (PRE006)*

		• *'I told you that two weeks ago you just dragged the imminent so that just feels, sometimes you are frustrating but then like I said, at the back of my mind you are also aware of the fact that that is the reality of life, as well.' (PRE005)*<br>• *'There are now so many times I have had, I have literally when I felt this patient should have been on end of life I had literally gone and asked the consultant I said are you just prolonging the life or are you just delaying the imminent.' (PRE005)*
Environmental context and resources	Guidelines and tools to support care	• *'...we have standard end of life care pathway that we use. We don't have a specialised stroke specific end of life care pathway... there isn't any option of yes the patient will deteriorate over time and may die in 6 months, that is not an option in that end of life care pathway.' (PRE009)*<br>• *'There is nothing that we use that guides us on that decision making process. It is usually just a team decision between the consultants and the nurse practitioners and the rest of the ward medical staff' (PRE010)*
	Lack of external resources	• *'If those patients are going to care homes, that is easier. Although the availability of beds at the minute is very dire. But people to their own home for example is much more difficult... Certainly because there is not the care, there is just not the care there for them. If it is a big family who are saying they are going to do everything, then actually our OT team can pull it together quite quickly with equipment and so on but they are, they are few and far between.' (PRE007 & PRE008)*
	Organisational model of care	• *'there has been a bit of that not understanding that it is not coming from us we have to follow policies by government, and NHS.' (PRE013)*
	Resources for staff training	• *'there is eLearning for palliative care, erm... but apart from that there is nothing really regular that happens.' (PRE001)*<br>• *I am not aware of any sort of formal training that you can sign up for like a half study day or a couple of hours, or a Teams meeting or*

		whatever where you could learn about palliative care and care of the dying which would certainly be useful.' (PRE010)
	Staffing levels	• *'the staffing levels have been quite trying. We have a lot of erm... bank or agency workers and some of them are fabulous, and some of them have not a scooby. So, erm... I think that's the hardest thing, especially for end of life care, is getting across why these patients need seeing so often and to make sure that you don't wait too long before you go in and see how they are doing.' (PRE013)*<br>• *'although we are quite a small stroke team, I think we are, I feel that the core of us are very committed and I think that we all erm... really care about our patients and our job and us as a team and that we want to kind of deliver the best care that we can with what we have. I think that it is finding ways to sort of erm... explore, expanding our kind of resources really in order to make sure that we do deliver that.' (PRE011)*
	Environmental resources	• *'I think we need something that I quite streamline, that is not going to take up a lot of time because we don't have a lot of time to spare. It is a busy ward, there is a lot of things happening, a lot of admissions, discharges, unwell patients, unfortunately at the moment there is staffing issues so it has to be relatively streamlined otherwise it is going to cause more problems than benefits' (PRE013)*
Social professional role & identity	Consultants assumed to be decision makers	• *'once an end of life decision has been taken, again like I said it is not just the consultant who makes that decision with the nursing team, the therapist, all of them we have an MDT meeting and once we have decided that the patient is put on end of life we do refer to this end of life team' (PRE005)*
	Requires collaboration & consensus	• *'I need to make sure that I am meeting with the family, I need to understand that family. I need to give them an update I need to make sure they understand their family prognosis. Then you walk away feeling quite good because the family understands, it gives them that opportunity to come to terms with it, it gives you that opportunity to make onward referrals to the palliative team, even to the psychology team.' (PRE002)*

		• *'people with a lot of expertise in recognising symptoms in a dying patient being reviewed by the right people' (PRE004).*
	Palliative Care lead	• 'I asked for like one CNS from the palliative care team, to be responsible for the stroke unit so at least we get to see you know the same person. But towards the end we had 4 different ones coming up to the unit. So that didn't help at all.' (PRE001)
Intentions	Timely conversations & decisions	• *'But I would like to think that we would provide them the best possible care regardless of when the decision was made that they would be palliative or dying if that makes sense […] Well we always try and have a conversation with family members and/or the patient if they are alert and awake enough' (PRE010)*
Social influences	Emotional impact upon staff due to sad nature of death	• *'…and all of a sudden unexpectedly, even for the nursing staff, that somebody dies like that it is just horrific for the, and we don't know them, know them like some of their relatives do.' (PRE010)*<br>• *'…I know that nurses tried their upmost and best because I think there is a lot of humanity in the team, erm… I am sorry to feel so strongly about that. I just think it was, quite devastating really for us as a service, especially with stroke because there is that sudden trauma and erm… I don't think that that is I just don't think that that is the way forward at all and I, I don't, I don't know…' (PRE011)*
	Impacts due to systems of care	• *'we still talk about it now you know how that all went wrong but a lot of that was erm… yes it was just that the patient wasn't reviewed erm… you know although the nurses were kind of like doing the right things, that you know, that case will always stick with me' (PRE001)*<br>• *'So it feels somewhere it feels very wrong to do that, ok because the patient is on end of life that is not my problem anymore. Because there are other patients who will benefit from coming to stroke, while logic explains that, it just feels wrong to do that as well.' (PRE005)*
	Affirmation from experts	• 'I think, we would benefit from a kind of like a strong voice that would erm… help to raise attention to that, and that might be a national drive or it might be a more local drive I don't know but I think that

		there needs to be some sort of drive there to recognise that. And bring it to our agenda you know.' (PRE011)
Emotion	Provision of formal emotional support	• *'...when they are dying and when they have cared for them for a couple of weeks and then suddenly somebody has a cardiac arrest and they die, it is a very emotional time for the nurses. So then you know just make them sit and talk them through it and all of that support. So we have given that, that again is ad hoc basis but we don't have such any officially kind of prescribed ok if somebody dies in your care that this, yes they know that we are there for them.' (PRE005)*<br>• *'even they come and do group sessions which I think was wanted more than it was actually used, once it came, erm... people then felt a little bit awkward I think. But erm... they are amazing, they are absolutely amazing what they offer. Erm... and we know that, as managers we can tap into that at any point if we were to ring them now and say our staff are struggling they would pull out all the stops to get here. So they are great and I think that is very much down to the people who are employed, the people who are in that role at this time they care a lot. They are very good.' (PRE007 & PRE008)*<br>• *'There are supposed to be a psychologist, a psychologist that comes around. There is supposed to be you know an occupational health department, that doesn't happen in practicality actually. It doesn't actually happen. People are supported by each other. I have had many incidents where certain staff have broken down, and they have been emotional, it is just us together, that erm... comfort them but I don't have, we don't have a network that helps.' (PRE009)*
	Supporting family emotion	• *'two of us there because I think it just helps to facilitate. Because even if there is more than one family member even and there are different emotions in the room and it just helps to sort of manage the situation more effectively.' (PRE011)*<br>• *'it is all about communication skills and the language you use and if you use, I am going to put your mum on a pathway, it sounds very horrible, very inhumane.' (PRE004)*

Skills	Interpersonal skills	• *'...I would first try to explain what has happened. For example if they have, the last one I had a discussion with was brain stem stroke, so I explained to them that you know he has had a stroke in a very sensitive area, which is responsible for a lot of major functioning in your body and if somebody does, that is not working then there isn't any means of survival and that is how you erm... then explore the possibility of end of life with those patients.' (PRE009)*<br>• *'...the palliative team are really good at supporting the nurses and obviously having those conversations with family about this is what it is going to look like from now on.' (PRE003)*
	Training other (in PC)	• *'It takes a lot of skill, it takes a lot of emotion, a lot of energy and you have got to have the right people who are able to deliver that message. Yes. Yes. So education is we definitely need education.' (PRE006)*<br>• *'...training and knowledge didn't have a place in terms of reducing the learning curve but key questions how you learn, how you the problem with end of life communication skills is you often have to do it to get it right. And you can listen to someone else doing it, yes, yes, I can do that, but in the circumstance, it is slightly different but, the other thing is observing people how do you communicate the decision, what is the language you use.' (PRE004)*<br>• *'...if we had more training around erm... end of life care, looking at different scenarios, erm... I think that would be amazing.' (PRE006)*
	Experiential Learning	• *'...it comes with experience if you are taught that. So actually if you don't have the education or somebody that is really good at doing care of the dying, and looking after families that has trained you, actually again you know because a lot of what we do nursing wise on the job is from peer learning...' (PRE010)*
Knowledge	Family knowledge	• *'Aphasic patients aren't able to tell you anything about what is going on, what they would like to happen you will have to talk to the family and get their views.' (PRE009)*

	Knowledge around nearing death	• *'senior nursing staff I you know they have as much knowledge about patients dying or not dying as anyone else. They have seen this all.' (PRE009)*<br>• *'…majority of people that come in erm… and die from their stroke may die sometimes from an aspiration, sometimes from raising of the cranial pressure, but more than likely you know it will be a combination or one or the other, erm… but either way their neurology is going to get worse, and they are going to slowly go down hill' (PRE001)*<br>• *'…what happens when you put someone on end of life care, they can have periods of slightly ups and downs especially at the end if the patient has got severe oedema put them on end of life care, oedema settles down and they have a brief awakening and if you explain that to them they know what to look out for and they know this is not, doesn't mean some one is getting better. But when that is not done they can mistake it for someone improving, not end of life.' (PRE004)*
	Lack of knowledge	• *'…if there was something more formal for people to follow, it would be easier for those less experienced staff members to know what to do.' (PRE011)*
	Procedural knowledge	• *'…, it is just second nature if you know what I mean. If you have got somebody who is palliative then we know that we have to go in so many times a day and check that they are comfortable, change their position, make sure they have got like the right mattress, the right erm… [14.42] make sure the families are in, the families are up to date, erm… it is also in the STARS(?) guidelines, the NICE guidelines on how to deal with palliative patients.' (PRE013)*
	Stroke specialist knowledge	• *'It is very variable. Sometimes if things, things don't always work for example some patients might, might die very acutely faster than you expected them to before you had a chance to discuss everything with the family. And some patients, may recovery much more than you would first anticipated.' (PRE009)*<br>• *'…some of these patients, they don't have anything medical going on they are not septic, their blood pressure in their boots etc, so it is*

		[43.57] withdrawal of feed, the time it takes for them to, and I explain to family ok, this is a different withdrawal compared to someone who has got unmeasurable blood pressure and [44.09: withdrawing] care and also I alluded to the point about severe oedema patients we see that repeatedly at times so, that kind of differences in the stroke patients.' (PRE004)<br>• *'…one of the things that, tools that guide us is our stroke scales itself. So for example the NIHSS tool that we have for stroke which allow us to guide towards the severity of the stroke, you know the score, the larger the score the higher the score the severe the stroke. So that is one tool. For ischaemic strokes as well we have something called the ICH score which basically looks at the volume of the blood in the brain […] we don't make a decision based on that on its own, we also take into account the premorbid, well prestrike condition of the patient, their baseline abilities, modified ranking score, how many other comorbidities were there along with the stroke, so sometimes there are 15 other things going on and the stroke may not be the biggest stroke it may be the last, or the tiniest of the stroke, but that tiny stroke probably was the last straw on the camel's back kind of a picture […] there are some cases which also tells us that the stroke itself was not big enough to cause the mortality but the whole picture when you take into account the patient that probably has contributed to the death as well.' (PRE005)*
	Training	• *'I already talked about to the point of deciding someone, this is the decision making process around saying someone is dying and you know, once you do that, I mean I must say that I haven't had any formal training on the erm… care pathway even though I was involved from the erm… [12.28] screens for translating to paper form into, I remember the erm… the Liverpool pathway, the withdrawal of it and the controversy around it, and how kind of paternalistic that was.' (PRE004)*<br>• *'…we encourage not just like the nurses, the auxiliaries, student nurses, doctors, therapy staff to do these erm… this STARS [15.27] to*

		work here, and then obviously the doctors, medical staff and the nursing staff have to do the advanced ones as well.' (PRE013)
	Achieving a good death	• *'...us as a medical team not intervening too much when we know that, those interventions are futile. And that is my idea of a good death where we allow that person to die with dignity, rather than intervening[...]symptom control, a comfortable patient who is well comfortable, family is around and allowing family to stay overnight, allowing extra family to come where they can have that period where they see that family member alive and having that whether it is their religion, having people come in all that for me is a dignified death.' (PRE002)*<br>• *'I think a good death is one which is identified in a timely manner. Explained to family member, in a timely manner and in the correct language and after a decision there is an ongoing review process to optimise comfort and early choice given in terms of place of care. More respecting a patient's wishes in terms of what they have, conducive with their wishes.' (PRE004)*
Goals	Consensus	• *'so we do have a MDT meeting and we do erm.. assess the patient's capacity, they look at their best interest and you know look at the whole, the bigger picture erm... and as a group then make a decision.' (PRE006)*
	Consistency	• *'some of the challenges that the nurses face could be alleviated by erm... medical decisions being done quite quickly and what should I say and the decisions being stuck to in a sense.' (PRE002)*
Behavioural regulation	Behavioural regulation when talking to families	• '...there is that fear around it of as you said before, like not wanting to say the wrong thing, or not wanting to put themselves out there in or in an opportunity to get do something wrong if you know what I mean like they don't, you know are they doing it right after death.' (PRE010)<br>• '...the ones that you know take a bit longer to accept it, the medical staff are usually pretty good at you know, meeting them regularly obviously the nursing staff kind of prompt that. We speak to them

		daily, erm... but it is important for us to have family involvement and erm... it is comforting for the patients to have their family with them.' (PRE013)
	Behavioural regulation with colleagues about personal opinions of care	• *'...I wouldn't want obviously you know, we might have had a very difficult erm... time and you can't be arguing with your colleagues about what you think is best for a patient.' (PRE001)*
Reinforcement	Positive reinforcement	• '...for the doctors give them more confidence around their prescribing. Obviously they are used to cautiously prescribing whereas the palliative care team are used to working in bigger doses and I think will often give them the advice and the encouragement to use actually what the patient does need.' (PRE007 & PRE008)
	Reinforcement by policy & guidance	• *'...it encourages you each day to identify any issues that you, any needs that you are not meeting. And erm... and put a care plan in place for that. And, then it asks you to go back and reflect on the outcome of that as well, how successful that's been.' (PRE007 & PRE008)*
Optimism	Optimism around quality of care	• *'...we are more frequently thinking we have done the best for that patient.' (PRE006)*<br>• *'We do pride ourselves on giving our palliative patients the best type of care.' (PRE013)*
	Optimism around teamwork	• *'...we have got a lot better at collectively thinking you know approaching a difficult situation as a team and the bereavement team being part of that.' (PRE006)*
	Pessimism	• *'...very, very large infarcts or very large bleeds don't tend to do overly fabulously and it is obviously it is an acute, quick change...' (PRE013)*

Supplementary Table S2 TDF domains and supporting quotes

Supplementary Table S1

How likely are the following groups to be involved in the decision-making process around end-of-life care for acute stroke patients?

	Highly likely %	Likely %	Unsure %	Unlikely %	Highly unlikely %	Missing %
Advanced Clinical Practitioner	16	21	5	10	7	42
Clinical Psychologist	3	4	2	16	31	44
Clinical Pharmacist	6	15	5	17	38	20
Dietitian	7	28	3	25	21	15
Health Care Assistant	7	16	2	30	31	15
Junior Doctor (Foundation and Core)	22	52	2	11	4	9
Mid-Grade Doctor	50	39	0	1	0	10
Occupational Therapist	12	35	1	31	10	11
Palliative Medicine and/or Eolc Consultant	23	28	2	24	9	14
Palliative Medicine and/or Eolc Nurse Consultant	27	25	2	20	6	21

Palliative and/or Eolc Team Member (Other Than Consultant/Nurse Consultant)	27	30	4	16	7	16
Patient	25	37	3	21	3	12
Physician Associate	8	16	9	12	11	45
Physiotherapist	13	37	1	26	13	11
Registered Nurse	45	40	0	3	2	9
Relative/Carer	65	24	0	2	1	8
Social Worker	3	11	2	33	31	20
Specialist Stroke Nurse/Nurse Practitioner	31	38	1	11	4	15
Speech And Language Therapist	16	44	1	20	10	9
Stroke Consultant	84	7	0	0	0	9
Stroke Nurse Consultant	15	11	1	6	6	61
Therapy Assistant	3	10	1	32	36	19
Other	2	1	0	0	0	97
